# Herausforderungen und Chancen für die chirurgische Weiterbildung: Ein fachgesellschaftsübergreifendes Positionspapier vor dem Hintergrund der Krankenhausstrukturreform

**DOI:** 10.1007/s00104-024-02113-x

**Published:** 2024-06-12

**Authors:** Frederik Schlottmann, Sabine Drossard, Maria Dey Hazra, Beate Blank, Marit Herbolzheimer, Joscha Mulorz, Juliane Kröplin, Tobias Huber, Panagiotis Doukas, Najla Sadat, Miriam Rüsseler, Romina Rösch, Frederic Bouffleur, Sarah Lif Keller, Gerrit Freund

**Affiliations:** 1 Junges Forum der Deutschen Gesellschaft für Plastische, Rekonstruktive und Ästhetische Chirurgie (DGPRÄC); Klinik für Plastische, Ästhetische, Hand- und Wiederherstellungschirurgie, Medizinische Hochschule Hannover, Carl-Neuberg-Str. 1, 30625 Hannover, Deutschland; 2 Arbeitskreis kinderchirurgischer Assistent*innen der Deutschen Gesellschaft für Kinderchirurgie (DGKCH), Klinik und Poliklinik für Allgemein‑, Viszeral‑, Transplantations‑, Gefäß- und Kinderchirurgie, Universitätsklinikum Würzburg, Oberdürrbacher Str. 6, 97080 Würzburg, Deutschland; 3 Junges Forum O und U der Deutschen Gesellschaft für Orthopädie und Unfallchirurgie (DGOU) und des Berufsverbands für Orthopädie und Unfallchirurgie (BVOU), Orthopädische Privatpraxis Dr. Kuhlee, Hönower Str. 16, 12623 Berlin, Deutschland; 4 Perspektivforum Junge Chirurgie der Deutschen Gesellschaft für Chirurgie (DGCH), Handchirurgie, Plastische und Mikrochirurgie, Dr. Erler Kliniken, Kontumazgarten 4–19, 90429 Nürnberg, Deutschland; 5 Junges Forum O und U der Deutsche Gesellschaft für Orthopädie und Unfallchirurgie (DGOU) und des Berufsverbands für Orthopädie und Unfallchirurgie (BVOU), BG Unfallklinik Murnau, Prof.-Küntscher-Str. 8, 82418 Murnau am Staffelsee, Deutschland; 6 Junges Forum der Deutschen Gesellschaft für Gefäßchirurgie und Gefäßmedizin (DGG), Klinik für Gefäß- und Endovaskularchirurgie, Universitätsklinikum Düsseldorf, Moorenstr. 5, 40225 Düsseldorf, Deutschland; 7 Perspektivforum Junge Chirurgie der Deutschen Gesellschaft für Chirurgie (DGCH), Klinik für Mund‑, Kiefer- und Plastische Gesichtschirurgie, Universitätsmedizin Rostock, Schillingallee 35, 18057 Rostock, Deutschland; 8 Chirurgische Arbeitsgemeinschaft Junger Chirurgen (CAJC) der Deutschen Gesellschaft für Allgemein- und Viszeralchirurgie (DGAV), Klinik für Allgemein‑, Viszeral- und Transplantationschirurgie, Universitätsmedizin der Johannes Gutenberg-Universität Mainz, Langenbeckstr. 1, 55131 Mainz, Deutschland; 9 Junges Forum der Deutschen Gesellschaft für Gefäßchirurgie und Gefäßmedizin (DGG), Klinik für Gefäßchirurgie, Universitätsklinikum Aachen, Pauwelsstr., 52074 Aachen, Deutschland; 10 Junges Forum der Deutschen Gesellschaft für Herz‑, Thorax und Gefäßchirurgie (DGTHG), Klinik für Herz- und thorakale Gefäßchirurgie, Universitätsklinikum Schleswig-Holstein-Campus Lübeck, Ratzeburger Allee 160, 23562 Lübeck, Deutschland; 11 Chirurgische Arbeitsgemeinschaft Lehre der Deutschen Gesellschaft für Chirurgie (DGCH), Klinik für Unfall‑, Hand- und Wiederherstellungschirurgie, Universitätsklinikum Frankfurt, Theodor Stern Kai 7, 60590 Frankfurt am Main, Deutschland; 12 Junges Forum der Deutschen Gesellschaft für Thoraxchirurgie (DGT), Abteilung für Thoraxchirurgie, Thoraxklinik Heidelberg der Universitätsklinik Heidelberg, Röntgenstraße 1, 69126 Heidelberg, Deutschland; 13 Junges Forum der Deutschen Gesellschaft für Mund‑, Kiefer- und Gesichtschirurgie (DGMKG), Klinik für Mund‑, Kiefer- und Gesichtschirurgie, Universitätsklinikum Heidelberg, Röntgenstraße 1, 69126 Heidelberg, Deutschland; 14 Junges Forum O und U der Deutsche Gesellschaft für Orthopädie und Unfallchirurgie (DGOU) und des Berufsverbands für Orthopädie und Unfallchirurgie (BVOU), Unfall‑, Hand- und Orthopädische Chirurgie, Städtisches Klinikum Karlsruhe, Moltkestraße 90, 76133 Karlsruhe, Deutschland; 15 Junges Forum der Deutschen Gesellschaft für Plastische, Rekonstruktive und Ästhetische Chirurgie (DGPRÄC), Klinik für Plastische Chirurgie, Hand- und Verbrennungschirurgie, Universitätsklinikum Aachen, Pauwelsstr., 52074 Aachen, Deutschland; 16 Klinik für Plastische, Ästhetische, Hand- und Wiederherstellungschirurgie, Medizinische Hochschule Hannover, Carl-Neuberg-Str. 1, 30625 Hannover, Deutschland

**Keywords:** Weiterbildung, Deutschland, Medizinische Ausbildung, Gesundheitsreform, Ambulantes Operieren, Internship and residency, Germany, Medical education, Healthcare reform, Outpatient surgery

## Abstract

**Hintergrund:**

Die chirurgische Weiterbildung steht bereits jetzt vor erheblichen Herausforderungen. Durch die geplante Krankenhausstrukturreform kommen neue bürokratische und organisatorische Hürden hinzu, die zu einem erheblichen Qualitätsverlust der chirurgischen Weiterbildung führen können.

**Fragestellung:**

Das vorliegende Positionspapier beschreibt aktuelle und zukünftige Herausforderungen für die chirurgische Weiterbildung und identifiziert mögliche Ansatzpunkte und Chancen für ihre Weiterentwicklung vor dem Hintergrund der geplanten Krankenhausstrukturreform.

**Material und Methoden:**

Für die Erarbeitung dieses Positionspapiers wurden durch ein Gremium aus Vertreterinnen und Vertreter der Jungen Foren der Deutschen Fachgesellschaften der chirurgischen Fächer aktuelle Probleme und Herausforderungen des derzeitigen Weiterbildungssystems identifiziert, kritisch diskutiert und ein Forderungskatalog für ein zukunftsfähiges Weiterbildungskonzept formuliert.

**Ergebnisse:**

Die geplante Ambulantisierung und Zentralisierung wurden als zentrale Herausforderungen für die chirurgische Weiterbildung identifiziert. Die ärztliche Weiterbildung muss bei allen Reformbestrebungen konsequent und von Anfang an mitgedacht werden. Neben einer transparenten und aufwandsgerechten Finanzierung der Weiterbildung fordern wir die Einbeziehung der Fachgesellschaften sowie eine Beachtung der sozialen Rahmenbedingungen für den chirurgischen Nachwuchs.

**Schlussfolgerungen:**

Der von der Politik forcierte Strukturwandel der Krankenhauslandschaft in Deutschland birgt die Gefahr, dass es zu einem weiteren Qualitäts- und Erfahrungsverlust in der chirurgischen Versorgung und Weiterbildung kommt. Gleichzeitig bietet das geplante Reformvorhaben aber die einzigartige Chance, bestehende Probleme aufzugreifen und die chirurgische Weiterbildung zukunftsfähig weiterzuentwickeln.

## Abstract (en)

**Background:**

Even now the further training in surgery faces considerable challenges. The planned hospital structural reform will result in new bureaucratic and organizational hurdles, which could lead to a considerable loss of quality in advanced surgical training across all disciplines.

**Objective:**

The aim of this position paper is to describe the current and future challenges for advanced surgical training and to identify possible approaches and opportunities for the further development against the background of the planned hospital structural reform.

**Material and methods:**

For the development of this position paper a committee of representatives of the Young Forums of the German surgical societies identified and critically discussed current problems and challenges of the present residency training system and formulated a list of demands for a sustainable residency training concept.

**Results:**

The planned shift to outpatient treatment and centralization were identified as central challenges for surgical residency training. Surgical training must be considered consistently and from the outset in all political reform efforts. In addition to a transparent and cost-appropriate financing of residency training, we call for the involvement of all German surgical societies in the reform process. Furthermore, the social framework conditions for junior surgeons should be considered.

**Conclusion:**

The structural change in the hospital landscape in Germany, which is being forced by politicians, harbors the risk of a further loss of quality and experience in surgical treatment and training. At the same time, the planned hospital reform offers a unique opportunity to address existing problems and challenges in surgical training and to consider them as a starting point for structural changes which are fit for the future.

Die derzeitige Situation der Weiterbildung stellt angehende Chirurginnen und Chirurgen sowie die Weiterbildungsermächtigten vor Herausforderungen aufgrund von Strukturproblemen und Ressourcenmangel. Vor dem Hintergrund der politischen Bestrebungen der Krankenhausstrukturreform befürchten die Autorinnen und Autoren fächerübergreifend einen erheblichen Qualitätsverlust der chirurgischen Weiterbildung. Gleichzeitig bietet das Reformvorhaben die einzigartige Chance, bestehende Probleme und Herausforderungen aufzugreifen und als Ausgangspunkt für einen Strukturwandel zu betrachten.

Laut der Ärztestatistik 2022 der Bundesärztekammer (BÄK) bleibt seit drei Jahren das Wachstum der Zahl an Ärztinnen und Ärzten in Deutschland hinter den Erwartungen zurück. Im Jahr 2022 waren insgesamt 421.252 Ärztinnen und Ärzte in Deutschland berufstätig, davon 40.766 in der Chirurgie. Von diesen waren 13.099 im ambulanten Bereich und 24.801 im stationären Bereich tätig. Darüber hinaus stieg die Zahl deutscher Ärztinnen und Ärzte, die in das Ausland abwanderten von 1081 auf 1301, was einer Zunahme von 20,4 % entspricht [2]. Während viele Chirurginnen und Chirurgen der älteren Generationen in den kommenden Jahren in Rente gehen, fällt es vielen Kliniken zunehmend schwerer, motivierte Ärztinnen und Ärzte für die chirurgische Weiterbildung zu gewinnen. So gaben in einer Umfrage unter deutschen chirurgischen Kliniken 80 % der Befragten an, einen spürbaren numerischen, in 94 % sogar einen qualitativen Bewerbermangel wahrzunehmen. 88 % der Befragten gaben an, dass sich der Bewerbermangel negativ auf die Versorgungsqualität auswirke [14]. In einer Umfrage unter Medizinstudierenden der Universität Münster zeigten 21 % der Befragten im 1. Semester Interesse an einer chirurgischen Weiterbildung, hingegen nur noch 13 % im 9. Semester [13]. In einer Onlineumfrage aus dem Jahr 2022 in Deutschland, Österreich und der Schweiz zeigte sich, dass der Wunsch, einer chirurgischen Weiterbildung nachzugehen, im Verlauf des Medizinstudiums kontinuierlich abnahm: Gaben während der Vorklinik 35 % der Studierenden Interesse an einer chirurgischen Weiterbildung an, waren es während des klinischen Studienabschnitts noch 22,6 % und während des Praktischen Jahres nur noch 19 % [8]. Eine ältere Befragung aus dem Jahr 2017 ergab, dass sich 40–60 % der Studierenden im 1. Semester eine chirurgische Laufbahn vorstellen konnten, hingegen nach Abschluss des Praktischen Jahres nur noch eine einstellige Prozentzahl [15]. Eine zunehmende Exposition zum beruflichen Alltag scheint mit einer Abnahme des Interesses an einer chirurgischen Karriere einherzugehen, was eine problematische Ausgangslage für die chirurgische Weiterbildung in Deutschland bedeutet. Betrachtet man die aktuellen demographischen Daten sowie die Sichtweise der kommenden Generationen auf die berufliche Perspektive, so muss eine Neustrukturierung der chirurgischen Weiterbildung stattfinden, um einem Mangel an Fachärztinnen und Fachärzten (FÄ) in Zukunft entgegenzuwirken und die chirurgische Versorgung in Deutschland langfristig zu sichern.

Nach Abschluss des Medizinstudiums entscheiden sich viele Ärztinnen und Ärzte für eine Weiterbildung. Der gesetzliche Auftrag zur Weiterbildung basiert auf der Grundlage, dass erfahrene FÄ andere Ärztinnen und Ärzte weiterbilden und ist von den Bundesländern auf die Landesärztekammern (LÄK) übertragen worden. Die Weiterbildungszeit ist dabei zwingend bei einer Person zu absolvieren, die von der Ärztekammer eine Weiterbildungsermächtigung erhalten hat. Die aktuelle Musterweiterbildungsordnung (MWBO) sieht für die Facharztanerkennung in den chirurgischen Fächern eine Weiterbildungszeit von mindestens 72 Monaten vor, wovon jeweils 6 Monate in der Notaufnahme und auf der Intensivstation abzuleisten sind. Zudem wird ein umfassender Katalog an Operationen und praktischen Fertigkeiten gefordert, bevor eine Zulassung zur Facharztprüfung erfolgen kann. Ärztinnen und Ärzte in chirurgischer Weiterbildung (ÄiW), aber auch Weiterbildungsermächtigte sehen sich derzeit mit verschiedenen Problemen und Herausforderungen konfrontiert. Bereits heute besteht in der chirurgischen Weiterbildung eine erhebliche Arbeitsverdichtung, die gepaart mit einem zunehmenden Fachkräftemangel und einer Reduktion der Qualität der Weiterbildung letztendlich eine Gefährdung für die Patientenversorgung darstellt. Zum Erhalt einer guten medizinischen Versorgung ist es zwingend erforderlich, ausreichend Nachwuchs zu rekrutieren und das bestehende Personal durch eine Verbesserung der Arbeitsbedingungen langfristig zu halten. Den Ansprüchen und Wünschen der ÄiW muss dabei ebenso begegnet werden wie den Vorstellungen und Konzepten der Weiterbildungsermächtigten, der Weiterbildungsstätten sowie der Ärztekammern.

Für die Erarbeitung dieses Positionspapiers erfolgte die Zusammenstellung eines Gremiums aus Vertreterinnen und Vertretern der Jungen Foren der chirurgischen Fachgesellschaften Deutschlands, um die derzeitigen Problematiken und Chancen der chirurgischen Weiterbildung zu identifizieren und vor dem Hintergrund der geplanten Krankenhausstrukturreform zu diskutieren. Der von der Politik forcierte Strukturwandel der Krankenhauslandschaft in Deutschland birgt die Gefahr, dass es zu einem weiteren Qualitäts- und Erfahrungsverlust in der chirurgischen Versorgung und Weiterbildung kommt. Insgesamt befürchten wir fächerübergreifend einen erheblichen Qualitätsverlust der chirurgischen Weiterbildung. Gleichzeitig bietet das geplante Reformvorhaben die einzigartige Chance, bestehende Probleme und Herausforderungen der chirurgischen Weiterbildung aufzugreifen und als Ausgangspunkt für einen Strukturwandel zu betrachten.

## Probleme der aktuellen chirurgischen Weiterbildung

### Ambulante und stationäre Weiterbildungsstätten

Die chirurgische Weiterbildung wird von Weiterbildungsermächtigten im ambulanten und stationären Sektor durchgeführt. Dabei ist die Weiterbildungsermächtigung personen- und nicht klinikgebunden, so dass bei einem Wechsel der Abteilungsleitung eine Weiterbildungsermächtigung stets neu bei der zuständigen LÄK beantragt werden muss. Dieser bürokratische Akt nimmt in der Regel einen mehrmonatigen Zeitraum in Anspruch. Die abzuleistenden Inhalte der Weiterbildung werden von den LÄK vorgegeben, die basierend auf der MWBO der Bundesärztekammer (BÄK) eine für die jeweilige LÄK geltende Weiterbildungsordnung festlegen. Die Organisation der Weiterbildung erfolgt überwiegend in Eigenregie durch die ÄiW. Auch wenn zunehmend eine kompetenzbasierte Weiterbildung und strukturierte Kurrikula gefordert werden, erfolgt die chirurgische Weiterbildung in Deutschland aktuell weiterhin überwiegend arbeitsplatzbasiert und nicht strukturiert [5, 6]. Flächendeckend verbindliche Konzepte fehlen hier ebenso wie einheitliche Vorgaben und standardisierte Lernzielkontrollen, bspw. welche Fähigkeiten in welchem Weiterbildungsjahr erworben werden sollen. Auch die Art und Weise, wie die zu erlernenden Fähigkeiten vermittelt werden sollen, bspw. im Rahmen von Fortbildungen, Simulationsszenarien, Operationskursen o. Ä., werden bisher ebenfalls nicht vorgegeben. Die vermittelten Kompetenzen werden bei noch nicht flächendeckender Einführung des e‑Logbuches zum Ende des jeweiligen Weiterbildungsabschnittes durch die Weiterbildungsermächtigten abgezeichnet. Dabei wird laut aktueller Studienlage der überwiegende Anteil der chirurgischen Weiterbildung nicht durch die Weiterbildungsermächtigten selbst, sondern durch andere FÄ erbracht [11]. Ein Schlüssel von betreuenden FÄ zu ÄiW wird dabei ebenso wenig vorgegeben. Immer seltener ist aufgrund zurückgefahrener Kapazitäten und zunehmender Spezialisierung eine vollständige Absolvierung der gesamten Weiterbildungszeit von 72 Monaten in einer einzelnen Klinik oder Praxis möglich. Somit absolvieren viele ÄiW ihre chirurgische Weiterbildung auch in mehreren Weiterbildungsstätten. Hospitationen in anderen Kliniken und Praxen werden aufgrund fehlender Kooperationen oder arbeitsrechtlicher Hürden bisher überwiegend nicht standardisiert und regelhaft angeboten, sondern müssen durch die ÄiW eigenständig organisiert werden. Ausbildungsbezogene Rotationsmodelle nach internationalem Vorbild, wie bspw. in der Schweiz oder den USA, sind nicht etabliert. Gleichzeitig dürfen konventionelle Techniken und Inhalte nicht gänzlich außer Acht gelassen werden und müssen, sofern nicht in der Praxis abbildbar, durch Simulationen vermittelt werden.

In einer 2020 veröffentlichen Studie wurde gezeigt, dass in 24,4 % der Fälle ein Eingriff komplett von ÄiW durchgeführt wurde und in 22,2 % der Fälle einzelne Teilschritte durch ÄiW übernommen wurden. Somit erfolgte bei knapp der Hälfte der Indexoperationen keine operative Weiterbildung. Die Autorinnen und Autoren kamen hier zur Schlussfolgerung, dass anhand der Daten von einer geringen Beteiligung chirurgischer ÄiW im Operationssaal ausgegangen werden muss und plädieren für eine konsequentere Umsetzung des Teilschrittekonzeptes [7]. Eine Onlineumfrage unter gefäßchirurgischen ÄiW aus dem Jahr 2023 ergab, dass nur 39,5 % mit ihrer Weiterbildung zufrieden waren, während 30,7 % nur mäßig zufrieden waren. Unter den Befragten gaben 53 % an, dass strukturierte Weiterbildungskonzepte existierten [4]. Die Umfrage zeigte hierbei ein deutliches Übergewicht der Weiterbildung im Überregional- und Maximalversorgungsbereich. Bereits unter aktuellen Bedingungen besteht die Sorge unter den ÄiW, dass ein Erreichen der notwendigen Richtzahlen in der bisher üblichen Weiterbildungszeit im Rahmen der neuen Weiterbildungsordnung (2020) nicht möglich sein wird [12]. Unter Berücksichtigung der aktuellen Literatur zeigen sich die Ursachen für einen inadäquaten Einsatz der ÄiW im Operationssaal insbesondere in der überbordendenden Verrichtung administrativer und nichtärztlicher Tätigkeiten, fehlender Strukturierung der Weiterbildung bzw. mangelnder Adhärenz zu vorhandenen Strukturen und ungenutzter Ressourcen [9].

Eine zunehmende Ambulantisierung des chirurgischen Sektors stellt diese ohnehin schon schwierige Aufgabe vor eine zusätzliche Herausforderung. Laut Ärztestatistik 2022 sind nur 32 % der Chirurginnen und Chirurgen in Deutschland im ambulanten Bereich tätig [2]. Eine zunehmende Verlagerung der Patientenversorgung in den ambulanten Bereich bedeutet, dass eine Ausweitung der chirurgischen Weiterbildung im ambulanten Sektor zur Erfüllung der Weiterbildungsordnung unumgänglich sein wird. Unter Berücksichtigung der bereits bestehenden Defizite in der chirurgischen Weiterbildung ist zu befürchten, dass eine weitere Ambulantisierung die Situation weiter verschärfen wird. Eine Verlagerung von Krankenhausbehandlungen hin zu ambulanten Eingriffen, hier insbesondere elektiver Eingriffe, vor dem Hintergrund der deutlich geringeren Zahl ambulant tätiger Chirurginnen und Chirurgen, würde die Möglichkeit der Weiterbildung und das Erreichen der notwendigen Richtzahlen vermutlich künstlich verknappen. Im Hinblick auf eine Sicherstellung der Patientenversorgung würde dies ebenfalls mit deutlichen Einschnitten einhergehen.

### Finanzierung der Weiterbildung und rechtliche Aspekte

Derzeit findet die chirurgische Weiterbildung keine adäquate und ausreichende finanzielle Abbildung. Kliniken und Praxen erhalten keine zusätzliche, geschweige denn adäquate Vergütung, wenn eine chirurgische Weiterbildung angeboten wird. Dies erfasst das vorliegende Positionspapier als Kernproblem der Weiterbildung in Deutschland. Aufgrund einer zunehmenden finanziellen Ressourcenknappheit wird es für Kliniken oder Praxiseigentümer somit immer unattraktiver Zeit und Geld in die ressourcenintensive Weiterbildung von ÄiW zu investieren. Um die Qualität der Weiterbildung zu gewährleisten, sollten die Weiterbildungsermächtigten entsprechend ihrer Verantwortung für die Aus- und Weiterbildung anhand festgelegter Qualitätskriterien ausgewählt sowie speziell geschult und laufend evaluiert werden. Den Weiterbildungsermächtigten sollten gesondert ausgewiesene Mittel und geschützte Zeitkontingente zur Verfügung gestellt werden, um ihre Aufgaben in der Aus- und Weiterbildung zu erfüllen.

ÄiW erhalten befristete Arbeitsverträge, die selten die gesamte Weiterbildungszeit umfassen und gehen somit erhebliche private und finanzielle Risiken ein. Konkrete Zahlen diesbezüglich fehlen bisher in der Literatur. Ebenso werden durch befristete Arbeitsverträge Abhängigkeiten geschürt, welche die Arbeitsgestaltung sowie die Weiterbildung negativ beeinflussen können. Um diesen Problematiken entgegenzuwirken, ist eine Neuregelung der Weiterbildungsstruktur und Anpassung der Finanzierung notwendig.

Auch die rechtlichen und versicherungstechnischen Hürden zur regelhaften Durchführung chirurgischer Eingriffe im ambulanten Sektor sind derzeit als problematisch zu betrachten. Hier ist der Facharztstatus im ambulanten Sektor erfreulicherweise durch den Facharztstandard ersetzt worden, wodurch ambulante Eingriffe auch unter der Anleitung und Aufsicht von FÄ durchgeführt werden können. Zudem sind Standortwechsel zwischen dem stationären und ambulanten Sektor aktuell fast immer mit einem Wechsel des Arbeitgebers verbunden.

Es ist inakzeptabel, dass der Weiterbildung aktuell kein substanzieller finanzieller Stellenwert und damit keine angemessene Würdigung beigemessen wird.

### Weiterbildung vor dem Hintergrund des Föderalismus

Die chirurgische Weiterbildung ist in Deutschland durch die 17 regionalen LÄK auf Basis der Heilberufegesetze der Länder organisiert. Basierend auf der MWBO der BÄK ergeben sich dadurch 17 verschiedene Weiterbildungsordnungen mit unterschiedlichen Regelungen und Einschränkungen. Durch die föderale Organisation besteht ein träges System, das einem dynamisch erforderlichen Weiterbildungskonzept nicht nachkommt. Änderungen der MWBO können nur einmal jährlich auf dem Ärztetag beschlossen werden. Eine Umsetzung in die jeweiligen Weiterbildungsordnungen der LÄK zieht dann einen entsprechend langen Zeitraum nach Beschlussfassung nach sich, da zudem noch die Prüfung anderer Richtlinien erfolgen muss.

Erfolgt im Rahmen der Weiterbildung ein Wechsel zwischen verschiedenen LÄK, sehen sich die ÄiW häufig mit bürokratischen Hürden und fehlender Bereitschaft der Anerkennung geleisteter Weiterbildungsinhalte konfrontiert. Um eine bundeslandübergreifende Anerkennung der Weiterbildung ohne Verlust an Weiterbildungszeiten zu ermöglichen, bedarf es standardisierter Verfahren zur inhaltlichen Bewertung der absolvierten Weiterbildung sowie der erworbenen theoretischen und praktischen Fertigkeiten.

### Innovative Aspekte für die chirurgische Weiterbildung

Die fortschreitende Digitalisierung wird bisher unzureichend in der chirurgischen Weiterbildung abgebildet [3, 10]. Innovative diagnostische oder therapeutische Verfahren, wie Robotik oder minimal-invasive Eingriffe, Big Data Science und personalisierte Medizin sind bisher nicht in den Weiterbildungsordnungen vorgesehen. Aus der alltäglichen klinischen Versorgung sind diese Innovationen jedoch nicht mehr wegzudenken [12]. Auch im Hinblick auf die Lehrmethoden sind weder Kadaver- noch Simulationstraining, e‑Learning oder Virtual/Augmented Reality für den Kompetenzerwerb anerkannt. Insbesondere unter Berücksichtigung der rückläufigen offenen Operationsverfahren und der Progredienz der minimal-invasiven Operationen bieten Skills-Labs mit praktischen Übungen an Kadavern und Simulatoren ÄiW eine gute Möglichkeit, um Operationstechniken auch außerhalb des Operationssaals zu erlenen.

### Mangel an ÄiW

Die Weiterbildungsermächtigten sehen sich fächerübergreifend mit einem Mangel an qualifizierten und motivierten Bewerberinnen und Bewerbern konfrontiert, die eine chirurgische Karriere anstreben. Oft werden eine hohe Arbeitsverdichtung, ungünstige Arbeitsbedingungen und eine schlechte Vereinbarkeit von Familie und Beruf als Gründe angeführt [9]. Insbesondere unter Berücksichtigung der Geschlechterverhältnisse in der Medizin werden Kinderbetreuungsmöglichkeiten sowie die Fortführung der Weiterbildung in Schwangerschaft, Stillzeit und Elternzeit zu drängenderen Themen. Benötigt werden rechtliche Rahmenbedingungen für einen strukturellen Wandel zur Verbesserung der Vereinbarkeit von Erwerbstätigkeit und Care-Arbeit mit Schaffung von Unterstützungsangeboten. Der bereits vorherrschende Fachkräftemangel in den chirurgischen Fachgebieten wird sich in den nächsten Jahren durch das altersbedingte Ausscheiden älterer Generationen weiter verschärfen [2]. Es bedarf daher der Etablierung eines attraktiven Weiterbildungskonzeptes, um junge ÄiW sowie Studierende nachhaltig für eine chirurgische Karriere zu motivieren. Um weiterhin eine qualitativ hochwertige chirurgische Versorgung in Deutschland anzubieten, ist dies in den kommenden Jahren absolut unabdingbar.

## Herausforderungen der Krankenhausstrukturreform

### Ambulantisierung, Zentralisierung und Finanzierung

Im Rahmen der geplanten Krankenhausstrukturreform soll ein nicht unerheblicher Anteil an chirurgischen Eingriffen in den ambulanten Sektor verschoben werden. Hier sei beispielsweise auf zahlreiche Eingriffe der Schilddrüsen- und Hernienchirurgie sowie der Handchirurgie verwiesen. Hier besteht die Problematik einer bisher noch nicht vorhandenen umfassenden ambulanten Versorgungsstruktur. Zudem ist aufgrund einer Zentralisierung und Gruppierung der Kliniken nach Levels mit einer Verschiebung der chirurgischen Versorgung in größere Zentren zu rechnen. Inwieweit dies Einfluss auf die Finanzierung der Kliniken und damit der Weiterbildung haben wird, verbleibt derzeit unklar. Es ist jedoch mit einer zunehmenden Verschärfung der prekären Finanzierungslage der Kliniken zu rechnen. Derzeit berichtet eine Vielzahl von Kliniken von erheblichen finanziellen Problemen, ein noch größerer Anteil von einer unzureichenden Finanzierung. Laut aktuellen Erhebungen des Deutschen Krankenhausinstituts berichten 65 % der befragten Kliniken im November 2023 von einer schlechten bis sehr schlechten finanziellen Lage [1]. Es bedarf daher eines Finanzierungsmodells, das adäquat und angepasst die Versorgungswirklichkeit abbildet, um auch Weiterbildungsinhalte und deren Kosten korrekt abbilden zu können. Dies sollte transparent und unabhängig vom Träger der Weiterbildungsstätte ausgestaltet werden. Insbesondere die „kleineren chirurgischen Fächer“, wie die Plastische Chirurgie oder die Kinderchirurgie, werden hiervon erheblich betroffen sein und müssen bei der Reform unbedingt berücksichtigt werden, um die Versorgung in Zukunft sicherzustellen.

### Weiterbildung muss neu gedacht werden: Verbundweiterbildung

Die Abbildung der chirurgischen Weiterbildung ist in den geplanten Reformvorhaben unzureichend. Es besteht der knapp formulierte Wunsch der Empfehlungskommission nach der Etablierung von Verbundweiterbildungen, wobei die Weiterbildung durch ein Konsortium an Weiterbildungsermächtigten erbracht werden soll. Wie diese regionalen Verbünde aussehen sollen, verbleibt jedoch unklar. Hier sei auf die geschilderten Probleme des Föderalismus der LÄK sowie bestehende arbeits- und berufsrechtliche Hürden verwiesen. Die Ausgestaltung der Verbundweiterbildung muss mit hoher Priorität durch entsprechend qualifizierte Stellen erfolgen und darf nicht den derzeitigen Weiterbildungsermächtigten als Herkulesaufgabe zugewiesen werden. Es bedarf klarer struktureller Vorgaben und Änderungen durch die Politik, insbesondere um rechtliche Hürden zu überwinden und eine Planungssicherheit für ÄiW sowie die Weiterbildungsermächtigten zu schaffen. Gleichzeit dürfen bei der Etablierung von Verbundweiterbildungen auch soziale Faktoren, wie die Wohnortsgebundenheit der ÄiW, Anfahrtswege an andere Weiterbildungsstätten und Kinderbetreuungskonzepte nicht außer Acht gelassen werden. Die Einbindung der chirurgischen Fachgesellschaften in die Ausarbeitung und Umsetzung der Weiterbildungsordnungen ist daher zwingend erforderlich, um den Anforderungen und Wünschen sowohl der ÄiW als auch der Weiterbildungsermächtigten gerecht zu werden.

#### Infobox 1 Forderungen für die chirurgische Weiterbildung vor dem Hintergrund der Krankenhausstrukturreform

Basierend auf den oben geschilderten Problematiken und Herausforderungen wurde fachgesellschaftsübergreifend ein Forderungskatalog formuliert, der bei der Umsetzung der Krankenhausstrukturreform berücksichtigt werden sollte, damit eine langfristige und qualitativ hochwertige chirurgische Weiterbildung sichergestellt wird.

Finanzierung und rechtliche Rahmenbedingungen

- Unabhängige und transparente Finanzierung der Weiterbildung
- Ausreichende finanzielle und zeitliche Ressourcen für Weiterbildungsermächtigte, um ihre Aufgaben in der Aus- und Weiterbildung zu erfüllen
- Regelmäßige Überprüfung und Anpassung der Weiterbildungsordnungen und Abrechnungsmodelle, um moderne weiterbildungsrelevante Verfahren adäquat erlernen und finanziell substanziell abbilden zu können

Soziale Rahmenbedingungen für den chirurgischen Nachwuchs

- Flexible Arbeitszeiten in Voll- und Teilzeitmodellen
- Flächendeckender Ausbau von Kinderbetreuung, insbesondere in Betriebskindertagesstätten, passend zu den jeweiligen Arbeitszeitmodellen
- Schaffung von Arbeitsmöglichkeiten im Home Office
- Strukturierte und flächendeckend verbindliche Konzepte zum Operieren in der Schwangerschaft und Stillzeit

Organisatorische und didaktische Aspekte der Weiterbildung

- Einbindung der chirurgischen Fachgesellschaften in die Ausarbeitung und Umsetzung der Weiterbildungsordnungen
- Standardisiertes und strukturiertes Weiterbildungskonzept mit bundesweit einheitlicher Weiterbildungsordnung und -kurrikulum
- Integration von Digitalisierung und modernen diagnostischen und therapeutischen Technologien in die Weiterbildungsordnungen, bspw. Robotik, Simulationsszenarien und Virtual Reality
- Standardisierte und verpflichtende Kurskonzepte zum Erlernen theoretischer Kompetenzen
- Einbezug von Onlinelehre als arbeitsplatzunabhängige Möglichkeit des Kompetenzerwerbs im Rahmen der Weiterbildungskurrikula
- Qualitätssicherung der Weiterbildung durch Evaluation und Kontrolle der Weiterbildungsermächtigten
- Einbindung standardisierter didaktischer Fortbildungskonzepte für die Weiterbildungsermächtigten (Train-the-Trainer-Prinzip)

Ambulantisierung/Zentralisierung

- Etablierung von Verbundweiterbildungen
  - Rotationen und theoretische Kursangebote zur Erlangung einer umfassenden chirurgischen Kompetenz basierend auf einem standardisierten Weiterbildungskurrikulum
  - Organisation der Rotationen und Kursangebote durch den Arbeitgeber mit obligatorischer Kostenübernahme
  - Transparente Darstellung des zeitlichen Ablaufs des Weiterbildungskurrikulums
- Sektor- und klinikübergreifende Weiterbildungsverträge im Rahmen von Verbundweiterbildungen mit arbeitsrechtlicher und berufsrechtlicher Absicherung

#### Infobox 2 Dieses Positionspapier wird unterstützt durch

- Perspektivforum Junge Chirurgie (PFJC) der Deutschen Gesellschaft für Chirurgie (DGCH)
- Junges Forum der Deutschen Gesellschaft für Plastische, Rekonstruktive und Ästhetische Chirurgie (DGPRÄC)
- Junges Forum O und U der Deutschen Gesellschaft für Orthopädie und Unfallchirurgie (DGOU) und des Berufsverbands für Orthopädie und Unfallchirurgie (BVOU)
- Junges Forum der Deutschen Gesellschaft für Allgemein- und Viszeralchirurgie (DGAV)
- Junges Forum der Deutschen Gesellschaft für Gefäßchirurgie und Gefäßmedizin (DGG)
- Arbeitskreis kinderchirurgischer Assistent*innen (AkA) der Deutschen Gesellschaft für Kinderchirurgie (DGKCH)
- Junges Forum der Deutschen Gesellschaft für Mund‑, Kiefer- und Gesichtschirurgie (DGMKG)
- Deutsche Gesellschaft für Neurochirurgie (DGNC)
- Junges Forum der Deutschen Gesellschaft für Thoraxchirurgie (DGT)
- Junges Forum der Deutschen Gesellschaft für Thorax‑, Herz- und Gefäßchirurgie (DGTHG)

## Fazit für die Praxis

Der von der Politik forcierte Strukturwandel der Krankenhauslandschaft in Deutschland birgt die Gefahr, dass es zu einem weiteren Qualitätsverlust in der chirurgischen Versorgung und Weiterbildung kommt. Insgesamt befürchten wir fachgesellschaftsübergreifend einen erheblichen Qualitätsverlust der chirurgischen Weiterbildung. Gleichzeitig bietet das geplante Reformvorhaben die einzigartige Chance, bestehende Probleme und Herausforderungen der chirurgischen Weiterbildung aufzugreifen und als Ausgangspunkt für einen Strukturwandel zu betrachten. Bei der weiteren Ausgestaltung der Rahmenbedingungen der chirurgischen Weiterbildung bedarf es zwingend einer Einbindung der chirurgischen Fachgesellschaften. Neben der Schaffung adäquater finanzieller und personeller Ressourcen muss flächendeckend ein verbindliches Weiterbildungskurrikulum unter der Berücksichtigung innovativer Lehr- und Lernkonzepte geschaffen werden, um Weiterbildungsermächtigten und Chirurginnen und Chirurgen in Weiterbildung einen Rahmen für eine gute Weiterbildung zu schaffen, damit auch langfristig eine qualitativ hochwertige Patientenversorgung gewährleistet ist.
